# Pyorrhea Alveolaris*Read at the March meeting of the Seventh Councilor District Medical Society, Austin, Texas.

**Published:** 1908-10

**Authors:** Julian Smith

**Affiliations:** Austin, Texas


For Texas Medical Journal.

### Pyorrhea Alveolaris.*

BY JULIAN SMITH, D. D. S., AUSTIN, TEXAS.

The subject of treatment of pyorrhea alveolaris is one that is attracting more attention in the dental profession today than any other, and, as is evidenced by my appearing before you, by

*Read at the March meeting of the Seventh Councilor District Medical Society, Austin, Texas.

special invitation, to present a paper on this subject, it gratifies me to see the interest extending to our medical brethren. Oral hygiene should be a very important point of diagnosis in all pathological conditions, and pyorrhea in some of its various forms is the most prevalent of all disorders in the oral cavity. In order that a clear understanding of the theories of the causes of the trouble may be had, it will do no harm to refer somewhat to the histology of the affected parts. The pericementum or peridental membrane is the soft tissue occupying the space between the root of the tooth and the alveolar wall. It forms the connection between the tooth and the bone, and is continuous with the gum tissue. It is rich in its vascular supply, which obtains principally by anastomosis with the circulation in the gums, and has a work to perform which is vital to the tooth itself. Many of the diseases of the oral cavity have their origin in this membrane, while others start in the surrounding parts. It is here that pyorrhea is "born, bred and has its being." The disease is not a new one by any means, as, about thirty years ago, Dr. J. M. Riggs recognized it as a distinct lesion, and outlined a method of treatment, as a consequence of which the disease became generally known as Riggs' disease. The term pyorrhea alveolaris, by which name we now universally refer to its various forms, signifies a discharge of pus from the alveolar cavity, and such discharge is usually present, though not always. The etiology of the disease is still somewhat obscure, although quite a number of theories, each one having considerable merit, have been advanced. The question of whether it is a local trouble, or systemic, or both, all have their advocates, though from personal observation I incline to the latter theory.

The local causes of pyorrhea are mechanical irritation, such as overhanging fillings, badly adapted crowns, calcic and serumnal deposits, mechanical appliances for regulating, and crowded or irregular teeth. The systemic cause is autointoxication, and I will give in brief a description of how this condition is brought about, as described by one of our most original investigators on this subject.

There are at all times a variety of micro-organisms in the mouth ready to get busy at the moment that conditions are favorable. Such opportunities present themselves when a low state of vitality from whatever cause exists. The effects of nervous exhaustion, continual fevers, indigestion, hepatic insufficiency, serumnal calculus, constipation, uric acid diathesis and poor elimination.

Probably the most common cause in our Southern climate is hepatic insufficiency on account of malarial influences. According to Dr. Talbot, the secretions of the liver, the bile, acts in four different ways: First, it stimulates peristalsis in the small intestines; second, it regulates the degree of fermentation; third, it assists in emulsification and saponification of fat globules in the intestines, and fourth, it destroys putrefactive bacteria. The intestinal tract at all times contains bacteria, many of which are toxic. The poisons generated by these bacteria are gathered into the portal system and carried to the liver cells by the capillaries. For a time these cells can dispose of the toxins by destroying them, but if the stream flows faster than the capacity of the liver can exercise its disintoxicating functions, it becomes impaired, and the poisons pass on into the blood stream and hinder the proper cell metabolism of the entire body. These toxins or poisons in the blood affect the heart and cause a high blood pressure. The pericementum or peridental membrane being a richly nourished tissue and lying between two bone walls allows of no expansion of the arteries under this extra pressure, and the blood being filled with these poisonous products causes a congestion and strangulation of the arteries that produce a continuous mild form of irritation, and so lowers the vitality of the membrane that when met by one of the local conditions sets up an inflammation that is invaded by the bacteria present in the mouth, and destruction of the membrane follows. The action of arterial irritation from other causes mentioned are similar, especially poor elimination which is more prevalent in colder climates. The symptoms of the disease in its primary stage is not very apparent, as it is usually well developed before it is recognized. A great many cases diagnosed as pyorrhea are simple cases of interstitial gingivitis or inflamed conditions, due to calcic deposits, with no loss of tissue in the pericementum. When permissible of being diagnosed as pyorrhea, there is generally considerable loss of the peridental membrane with congested and hypertrophied arterial conditions at the most important part of the blood supply, viz., at the cervical margin of the gum. There are different phases of the disease which complicate the diagnosis, and which as yet have hardly been differentiated. Some begin and go on violently until the tooth becomes so sore and loose that it is lost in a few weeks; others progress very slowly, and take years to produce any serious trouble. Sometimes there is severe neuralgic pains, at other times the progress is so benign that the first intimation is the looseness of the tooth. And at times instead of the inflamed conditions there appears to be almost an anemic condition of the gums.

Contrary to what is generally believed, and as the name indicates, pus is not always present, though as a general thing, in its secondary stage this is the case. As to liability, we are all liable to have this disease, but conditions must be favorable. Secondary to local or mechanical irritation, we must have a susceptibility, a weakness. Dr. D. D. Smith, of Philadelphia, speaks of "any mouth with pyorrheic tendencies," evidently believing that what would be sufficient cause to produce pyorrhea in one mouth would not do so in another.

We find most of our cases among the people of a lymphatic temperament, sluggish circulation, absence of energy, who tire easily, etc. Next, those of a bilious temperament, with organic derangement. Then comes the nervous, overworked and overtaxed and lastly the sanguineous, who, however, is the exception rather than the regular pyorrhea subject. People of sedentary habits, such as lawyers, bankers, bookkeepers, etc., are usually the greatest sufferers.

There is no doubt in my mind that the disease can be contracted by one person from another, as is evidenced by the fact that I have never seen a case in a comparatively young patient where, upon inquiry, I did not find that either one or both parents had been sufferers from this trouble for a long time. Heredity does not enter into the question, except in so far as children inherit certain idiosyncracies or weaknesses, which might produce a susceptibility.

The deleterious effect of pyorrhea on the system can be better determined by the medical practitioner than by us, but the one item of a continual mixture in the food during mastication of millions of streptococci, to be carried into the stomach and there to be distributed to all parts of the system, if any escape their natural germicidal enemies, must have an evil effect on the vitality, and certainly give the organs that produce antitoxic agents an extra amount of work that might be put to better service.

The treatment of the disease from our standpoint, i. e., the dentist's, is purely a local one, and it is up to the medical profession to determine theirs. Thorough instrumentation, the removal of all foreign substances, all necrosed tissue from the root of the tooth and curetting the bone, leaving no ragged edges (due to absorption). Liberal bleeding of parts during operation, followed by thorough washing with some germicidal agent and calcic solvent, such as lactic acid, phenolsulphonic acid, 20 per cent solution trichloracetic acid, or aromatic sulphuric acid. The after-treatment consists of antiseptics, astringents and stimulants accompanied by frequent massage and a good aseptic mouth wash to be used continually.

In conclusion, I will say that if we could so thoroughly remove all foreign substances from the affected parts as to leave no nucleus for new deposits, we could declare our case cured until systemic conditions could reproduce similar causes that originated the trouble, but to say remove all foreign substances and to do it are two entirely different things. Prevention of contagion should be considered wherever the disease is recognized and a suitable aseptic mouthwash prescribed. Pyorrhea is a strong connecting link between our professions, for it is a menace to the health. In its treatment we need your services and you need ours, and the people need us both.

---

Frost Bites, or Chill Blains.—*L'Etoile médicale* (through *Bulletin général de thérapeutique*) extols the following method of treating frost bites, with a formula by Jadassohn. The feet are to be bathed from one to three times a day, in water as hot as can be borne, for a period of ten to fifteen minutes. They are then carefully dried, and, if the frost bites are ulcerated, they are washed with alcohol. In the evening the following ointment is used with prolonged massage, and a layer of it applied to the lesions. It consists of:

℞ Ichthyol ....................1.0 to 5.0 grammes
Resorcine ....................1.0 to 3.0 grammes
Purified wool fat..................55.0 grammes
Olive oil ........................10.0 grammes
Distilled water ..................50.0 grammes
M.

If the ulcers are extensive this ointment is to be applied twice daily. [A 3 per cent phenol ointment, made with petrolatum (unguentum phenolis, U. S. P.), applied without friction, has a local anesthetic effect and promptly relieves the irritation of chill blains, which are so annoying to children in winter.]

[From *"The Prescription."*]

